# Measuring respiratory symptoms in moderate/severe asthma: evaluation of a respiratory symptom tool, the E-RS®: COPD in asthma populations

**DOI:** 10.1186/s41687-021-00338-6

**Published:** 2021-10-10

**Authors:** Maggie Tabberer, Robyn von Maltzahn, Elizabeth D. Bacci, Hayley Karn, Ray Hsieh, Timothy A. Howell, Zelie Bailes, Andrew Fowler, Laurie Lee, Lindsey T. Murray

**Affiliations:** 1 GSK House, 980 Great West Road, Brentford, Middlesex, TW8 9GS UK; 2 Evidera, Patient-Centered Research, 615 2nd Avenue, Seattle, WA 98104 USA; 3 Evidera, Patient-Centered Research, 201 Talgarth Road, London, W6 8BJ UK; 4 Evidera, Patient-Centered Research, 7101 Wisconsin Avenue, Bethesda, MD 20814 USA; 5 GSK, 1250 S Collegeville Road, Collegeville, PA 19426 USA

**Keywords:** Asthma, Patient-reported outcome, Psychometric, Reliability, Validity, Responsiveness, Respiratory symptoms, Qualitative, Content validity

## Abstract

**Background:**

Symptom constructs included in the Evaluating Respiratory Symptoms in Chronic Obstructive Pulmonary Disease (E-RS®: COPD) tool may be relevant to patients with asthma. The purpose of this study was to evaluate content validity and psychometric performance of the E-RS: COPD in moderate/severe asthma patients.

**Methods:**

Content validity of the E-RS: COPD was evaluated in patients with moderate/severe asthma using concept elicitation and cognitive debriefing interviews. Secondary analyses using data from two clinical trials in patients with moderate/severe asthma evaluated the factor structure of the E-RS: COPD plus two supplementary items (wheeze; shortness of breath with strenuous physical activity) and assessed psychometric properties of the tool, which will be referred to as E-RS®: Asthma when used in asthma populations.

**Results:**

Qualitative interviews (*N* = 25) achieved concept saturation for asthma respiratory symptoms. Concepts in the E-RS: COPD were relevant to patients and instructions were understood. Most patients (19/25; 76%) reported experiencing all concepts in the E-RS: COPD; no patients indicated missing symptoms. Secondary analyses of clinical trial data supported the original factor structure (RS-Total and three symptom-specific subscales). The two supplemental items did not fit with this factor structure and were not retained. RS-Total and subscale score reliability was high (internal consistency [α] > 0.70). Validity was demonstrated through significant (*P* < 0.0001) relationships with the St George’s Respiratory Questionnaire (SGRQ) and Asthma Symptom Severity scale. E-RS: Asthma was responsive to change when evaluated using SGRQ, Patient Global Impression of Change and Asthma Quality of Life Questionnaire as anchors (*P* < 0.0001). Clinically meaningful change thresholds were also identified (RS-Total: − 2.0 units).

**Conclusions:**

The E-RS: Asthma is reliable and responsive for evaluating respiratory symptoms in patients with moderate/severe asthma.

**Supplementary Information:**

The online version contains supplementary material available at 10.1186/s41687-021-00338-6.

## Background

Asthma is a chronic inflammatory airway disease characterized by respiratory symptoms such as wheeze, shortness of breath (SOB), chest tightness, and cough [1], which impair health-related quality of life, often in proportion to disease severity [2–4]. Understanding overall symptom burden in patients with asthma is a critical step in assessing the impact of treatment-related changes in both clinical trials and clinical practice [2]. However, respiratory symptoms are often only assessed periodically using diaries in individual studies, making between-study comparisons difficult. This variation in methods used for symptom assessment indicates a need for improved patient-reported measures of symptomatic experience in asthma. Furthermore, asthma symptoms vary daily, indicating that a standardized daily diary measure is appropriate.

The Evaluating Respiratory Symptoms in Chronic Obstructive Pulmonary Disease (E-RS®: COPD) measure is an 11-item patient-reported diary derived from the 14-item The EXAcerbations of Chronic Pulmonary Disease Tool (EXACT®) [5]. The EXACT was developed to assess the frequency, severity, and duration of COPD exacerbations from the patient perspective. The E-RS: COPD assesses the cardinal respiratory symptoms of COPD as a total score through three symptom-specific domains: cough and sputum, chest symptoms, and breathlessness [6].

Literature reviews [7, 8] and qualitative research [9] indicate that respiratory symptom constructs in the E-RS: COPD may also be relevant to patients with moderate/severe asthma, as both are diseases of airflow obstruction and share common symptoms, such as SOB and cough [1, 10]. However, it is unclear whether the E-RS: COPD can adequately detect symptoms across the full range of asthma severity. Furthermore, no research has assessed content validity or psychometric performance of the E-RS: COPD in asthma populations.

The current analysis comprised a two-phase approach utilizing both qualitative and quantitative data. First, qualitative interviews were conducted to determine the suitability and content validity of the E-RS: COPD in a moderate/severe asthma population. Second, data from two clinical studies in moderate/severe asthma patients [11, 12] were used to evaluate the factor structure of the E-RS: COPD in asthma populations (hereafter referred to as the E-RS®: Asthma when used in the context of asthma patient populations). Subsequently, psychometric properties (ie, reliability, validity, and responsiveness) of the E-RS: Asthma were assessed in this patient population. A responder threshold for clinically important change in this asthma population was also established.

## Methods

### Phase 1: qualitative study

#### Study sample

A cross-sectional, qualitative study (GSK: 206605; ClinicalTrials.gov: NCT03344406) involving one-on-one semi-structured concept elicitation and cognitive debriefing telephone interviews was conducted between December 2017 and September 2018 with patients attending five US asthma and allergy clinics. Quorum Review Institutional Review Board (IRB) approval was obtained prior to initiation of the study (Quorum Review File # 32810). Interviews were conducted in English or Spanish language, lasted 60–90 min and were recorded and translated into English (if required). Eligibility criteria are summarized in Supplementary Table 1. Briefly, adults aged ≥18 years with moderate/severe asthma with a history of airflow obstruction and evidence of bronchodilator reversibility were eligible for inclusion. Current smokers and patients with a diagnosis of COPD or other clinically important lung conditions were excluded.

#### Procedures

Interviews followed a semi-structured interview guide. All participants provided written informed consent prior to the interview. Interview questions were used to elicit asthma symptoms from the patient perspective first, and subsequently participants completed the E-RS: COPD questions in a paper format. Participants provided further feedback during cognitive debriefing to assess comprehension, relevance, and completeness of the tool.

#### Analyses

Interviews were transcribed and anonymized prior to descriptive analysis using ATLAS.ti version 7.0 or higher (ATLAS.ti Scientific Software Development GmbH, Berlin, Germany). A coding dictionary was developed iteratively based on concept themes related to participants’ symptom experience. Words and phrases reported by interview participants were selected using the coding dictionary and grouped into key themes, attributes, concepts, and relationships. Saturation was defined as the point at which no major new themes, descriptions of a concept, or terms were introduced as subsequent interviews were conducted [13]. Concepts were subsequently thematically mapped to assess content coverage. Feedback on the E-RS: COPD was obtained using qualitative codes to capture overall feedback, item comprehension, recall period, and response options. Results are reported in accordance with the Consolidated Criteria for Reporting Qualitative Research (COREQ) checklist [14].

### Phase 2: quantitative psychometric evaluation

#### Study sample

Utilizing data from two randomized controlled trials (RCTs) (GSK: 205832; ClinicalTrials.gov: NCT03012061 [11]; GSK: 205715; ClinicalTrials.gov: NCT02924688 [12]), a quantitative psychometric evaluation was performed to first evaluate the factor structure of the E-RS: COPD with the intent to use this tool in a moderate/severe asthma population (E-RS: Asthma). Subsequently, the scoring algorithm and psychometric properties, including reliability, construct validity, and responsiveness were evaluated. IRB approval was obtained prior to initiation of patient recruitment or administration of measures as part of each clinical trial (205832 and 205715). No additional ethics committee or IRB approval were required for this secondary data analysis.

GSK 205832 was a Phase IIb, randomized, double-blind, placebo-controlled, three-arm, parallel-group study conducted between January 2017 and May 2018. The study evaluated the efficacy, safety, and tolerability of two doses of umeclidinium bromide (UMEC) administered once daily (QD) via the ELLIPTA dry powder inhaler (DPI) over 24 weeks in patients with moderate uncontrolled asthma receiving fluticasone furoate (FF) 100 mcg QD. GSK 205715 was a Phase IIIa, randomized, double-blind, active-controlled, six-arm, parallel-group study conducted between December 2016 and August 2018. This study compared the efficacy, safety and tolerability of four fixed-dose triple combinations of FF/UMEC/vilanterol (VI) with two fixed-dose dual combinations of FF/VI, administered QD via the ELLIPTA DPI for 24–52 weeks in patients with moderate/severe asthma uncontrolled on inhaled corticosteroids (ICS) ± a long-acting β_2_-agonist (LABA) therapy with the primary outcome of the study (clinic trough forced expiratory volume in 1 s [FEV_1_]) completed at Week 24.

Detailed eligibility criteria for both trials are summarized in Supplementary Tables 2 and 3, and have been presented elsewhere [11, 12]. Briefly, 205832 enrolled a moderate asthma population prescribed ICS ± a LABA or a long-acting muscarinic antagonist (LAMA), whereas 205715 included a broader population of patients with moderate/severe disease uncontrolled on ICS/LABA. There were also differences in asthma control at baseline. Thus, 205832 required patients to have Asthma Control Questionnaire (ACQ)-6 score > 0.75 at screening (partially or inadequately controlled asthma), whereas 205715 restricted entry to those only with inadequately controlled asthma symptoms (ACQ-6 score ≥ 1.5). Both studies required evidence of reversibility (post-bronchodilator increase in FEV_1_ of ≥12% and ≥ 200 mL following salbutamol inhalation), and evidence of airflow obstruction at screening, although this differed between studies at screening (e.g., pre-bronchodilator AM FEV_1_ < 85% and ≤ 90% predicted in 205715 in 205832, respectively). In 205715, patients were required to meet additional entry criteria at the end of the 3-week run-in period before entering the 2-week stabilization period and at the end of the stabilization period prior to randomization (Supplementary Table 3). In contrast, 205832 had a shorter 2-week run-in phase with patients required to meet additional criteria only prior to randomization (Supplementary Table 2).

#### Measures

Timing of collection of individual patient-reported outcomes (PROs) in both RCTs is summarized in Supplementary Table 4. All PROs used in 205832 and 205715 were administered on an electronic diary which was also programmed to allow collection of periodic PRO assessments during patient visits to study sites. Electronic administration of PROs, such as Asthma Quality of Life Questionnaire (AQLQ) and Asthma Control Questionnaire (ACQ) has demonstrated high levels of agreement with paper versions [15]. In addition, the US Food and Drug Administration (FDA) indicates that the St George’s Respiratory Questionnaire (SGRQ) can be administered electronically [16]. To avoid missing data, patients were required to provide a response before they could move to the next question. Once data were submitted, patients were unable to view their previous responses.

#### E-RS: COPD

The E-RS: COPD consists of 11 items that measure respiratory symptoms recalled by patients over the previous 24 h, rather than a change in symptoms over this timeframe, capturing information related to breathlessness, cough, sputum production, chest congestion, and chest tightness. Daily recording provides an assessment of underlying day-to-day variability of symptoms. Items 1–8 are scored on a 5-point scale of not at all to extreme, and items 9–11 are scored on a 6-point scale of not at all to too breathless to do these. Thus, the RS-Total score has a range of 0–40, comprising three subscales: RS-Breathlessness (sum of 5 items, range 0–17); RS-Cough and Sputum (sum of 3 items, range 0–11); and RS-Chest Symptoms (sum of 3 items, range 0–12) [6, 17]. For both the 205715 and 205832 studies, the E-RS was administered electronically with exactly the same appearance and format as the paper version administered in Phase 1.

#### Supplemental items

Alongside the E-RS: COPD, two supplemental items were included that were rooted in patient feedback from previously reported qualitative work [9]. First, a wheeze item was included, in which participants were asked “Did you wheeze today?” with response options of not at all, rarely, occasionally, frequently, and almost constantly. Second, a SOB with strenuous physical activity item was included with the question “Were you short of breath today when performing strenuous activities such as climbing stairs, running, or participating in sports activity?” with response options of not at all, slightly, moderately, severely, extremely, and too breathless to do these.

#### Patient Global Impression of Severity (PGI-S) and Patient Global Impression of Change (PGIC)

The PGI-S is a single-item questionnaire to evaluate disease severity; patients rated asthma symptoms they are currently experiencing at each study visit using a 5-point scale (none, mild, moderate, severe, very severe). The PGIC is a single question used to evaluate response to treatment since the start of the study using a 7-point scale (significantly improved, moderately improved, mildly improved, no change, mildly worse, moderately worse, significantly worse).

#### St George’s Respiratory Questionnaire (SGRQ)

The SGRQ is a measure of health status in patients with chronic airway obstruction [18], and includes 50 items addressing three domains: symptoms, activity limitations, and impact. Recall periods in the questionnaire include the past 4 weeks and the current day. A 5-point scale is used for rating symptoms and a true/false binary scale used for activity limitations. The total score is expressed as a percentage of overall impairment, with 0 and 100 representing the best and the worst possible health status, respectively. A reduction of ≥4 points in SGRQ total score is considered clinically meaningful [19].

#### Asthma Quality of Life Questionnaire (AQLQ)

The AQLQ includes 32 items that measure functional impairment related to asthma. The questions are designed to be self-completed by the patient, with a recall period of the past 2 weeks. The response scale ranges from 1 (totally impaired) to 7 (not at all impaired). A change of ≥0.5 is considered clinically important [20].

#### Asthma Control Questionnaire (ACQ-5, ACQ-6)

The ACQ measures various attributes of asthma control [21]. ACQ-5 includes five questions (nocturnal awakening, waking in the morning, activity limitation, SOB, and wheeze) that gauge the frequency and/or severity of symptoms over the previous week. The ACQ-6 includes an additional item relating to rescue medication use. The recall period is the past week. Response options range from 0 (no impairment/limitation) to 6 (total impairment/limitation). Scores < 0.75 indicate well-controlled asthma whereas scores ≥1.5 indicate poorly controlled asthma [22]. A change of ≥0.5 units is considered clinically important [23].

#### Analyses

Quantitative psychometric analyses used a statistical analysis plan informed by previous validation work on the E-RS: COPD in COPD [6] and in asthma–COPD overlap (ACO) syndrome [24]. Data were not pooled due to differences in study populations (Supplementary Table 5). This allowed for evaluation of psychometric properties across patients with moderate (205832) and moderate/severe asthma (205715).

Analyses were conducted on blinded data (205832) and interim blinded data (205715) from a PRO dataset, defined as patients included in the intent-to-treat populations with a minimum of 4 days of data for the week prior to baseline, using SAS statistical software version 9.4 (SAS Institute Inc., Cary, NC, USA) or STATA 15 (StataCorp LLC, College Station, TX, USA). All statistical tests were two-sided and used a significance level of 0.05. No imputation of missing data was performed.

#### Factor structure

Confirmatory factor analysis (CFA) was conducted using structural equation modeling to evaluate the fit of the factor structure of the E-RS: COPD in patients with moderate/severe asthma. The hypothesis that the E-RS: COPD has three factors and second order unidimensionality (ie, that the three factors load onto a single construct) was tested. First, the comparative fit index (CFI) evaluated the proportionate improvement in a model by comparing a hypothesized model against a less restricted baseline model [25]; values ≥0.9 indicate acceptable fit. Second, the standardized root mean residual (SRMR) measured the mean absolute difference between observed and model-implied correlations; values < 0.1 are considered acceptable [26]. Finally, the root mean square error of approximation (RMSEA) assessed the discrepancy between predicted and observed data per degree of freedom; values < 0.08 are considered acceptable [27]. CFA was estimated at Weeks 0 and 24 in both studies. Post hoc exploratory factor analysis (EFA) determined if there was an optimal factor structure, which includes the E-RS: COPD and the two supplemental items in a meaningful way, and was performed at Weeks 0 and 24 in both studies. In EFA, the structure or number of factors was not pre-specified; scree plots and corresponding eigenvalues were examined to determine the number of factors empirically [24]. The psychometric properties of the tool, referred to as the E-RS: Asthma when used in asthma populations, was then assessed using the best fitting factor structure.

#### Reliability

Reliability was assessed using internal consistency reliability and test–retest reliability. Cronbach’s alpha coefficient was estimated for the mean weekly RS-total and subscale scores at Week − 2 and at Weeks 0, 4, 12, and 24 (205832) and at Weeks − 5 and − 2 and at Weeks 0, 4, 12, 24, 36, and 52 (205715). Reproducibility of total and subscale E-RS: Asthma daily and mean weekly scores was assessed to evaluate test–retest reliability, utilizing intra-class correlation (ICC) coefficients with a two-way random effects regression model based on absolute agreement (ICC_2,1_) [28] and paired *t*-tests.

For daily scores, reproducibility of scores (test–retest) over consecutive days (Days 1–2, 2–3, 3–4, 4–5, 5–6, and 7–8) and over a 7-day interval (Days 1–7) from screen run-in indicated patients were stable during this period if randomized. For weekly scores, reproducibility of scores from the first to the second week of screen run-in were assessed. Additionally, reproducibility of scores for patients with no change in ACQ score at the Week − 2 visit (Visit 1 in 205832 and Visit 2 in 205715) to randomization (Visit 2 in 205832 and Visit 3 in 205715) were assessed.

#### Validity

Construct validity of the E-RS: Asthma total and subscale scores were assessed using convergent and discriminant validity and known-groups validity at Week 24. Convergent validity was considered supported if E-RS: Asthma scores showed moderate correlation (*r* > 0.40) with conceptually similar measures. A lower correlation (*r* < 0.40) was required to support discriminant validity. Spearman’s rank correlation coefficients were calculated between the mean weekly E-RS: Asthma score and mean weekly rescue medication use, expected peak expiratory flow (AM), expected FEV_1_ (AM), nighttime awakenings due to asthma symptoms, and asthma symptom severity scores. In addition, the mean weekly E-RS: Asthma score was compared with FEV_1_, FEV_1_% predicted, SGRQ (total and domain scores), AQLQ, and ACQ scores from baseline or final visit.

Known-groups validity of the total and subscale E-RS: Asthma daily and mean weekly scores was assessed using analysis of variance (ANOVA) by examining score differences during the baseline week in patients grouped according to FEV_1_% predicted categories, PGI-S, ACQ score and exacerbation history. The *F*-statistic from the ANOVA and the *t*-statistic from the *t*-tests were considered significant if both were below 0.05.

#### Responsiveness

Analysis of covariance (ANCOVA) models were used to examine differences in change in total and subscale E-RS: Asthma daily scores from baseline week to Weeks 4, 12, and 24, among patients in various responder groups. Baseline scores (mean of Day − 7 to − 1) were controlled for in the models. Cohen’s effect size was calculated for E-RS: Asthma total and subscale scores among patients defined as “responders” using PGIC, SGRQ, or AQLQ.

#### Responder threshold

Anchor and distribution-based methods were used to define a responder threshold for E-RS: Asthma. Using anchor-based methods, mean change in mean weekly E-RS: Asthma total and subscale scores from Weeks 0 to 24 were determined by PGIC level and assessed by plotting cumulative distribution function (CDF) graphs. The minimally important change threshold was the mean change in RS-Total and subscale scores of patients who were mildly improved or mildly worse on the PGIC. Exploratory analyses were conducted utilizing the SGRQ and AQLQ minimally important difference thresholds as anchors (change of 4.0 point and 0.5 points, respectively). Distribution-based methods were conducted as supportive information for development of a responder threshold and included an assessment of the standard error of measurement (SEM) and half standard deviation (SD). SEM was estimated by multiplying the baseline SD of the measure by the square root of one minus its reliability coefficient (ICC from the test–retest assessment) [29, 30]. Half an SD of a measure represents a good approximation of the minimally important difference [31].

## Results

### Phase 1: qualitative study

Twenty-five patients (24 English-speaking, 1 Spanish-speaking), identified from five asthma and allergy clinical sites around the USA, participated in concept elicitation interviews. Most participants were female (*n* = 15; 60%) and Caucasian (*n* = 21; 84%), with a mean (SD) age of 48 (16.5) years (range 23–83 years). Mean (SD) age of first asthma diagnosis was 21.3 (17.8) years (Table 1).

**Table 1:** Demographics and clinical characteristics in Phase 1 and Phase 2

Phase 1		
**Demographics**^a^		**Total (*N*** **= 25)**
**Age (years), mean (SD)**		48 (16.5)
**Female,** ***n*** **(%)**		15 (60.0)
**Racial background**^b^**,** ***n*** **(%)**		
White		21 (84.0)
Black or African American		3 (12.0)
Asian		1 (4.0)
American Indian or Alaska Native		2 (8.0)
Other		2 (8.0)
**Employment status**^b^**,** ***n*** **(%)**		
Employed full-time		18 (72.0)
Employed part-time		2 (8.0)
Retired		3 (12.0)
Student		1 (4.0)
Other		2 (8.0)
**Clinical characteristics**^c^		
**Time since diagnosis (years), mean (SD)**		10.8 (11.8)
**Asthma severity,** ***n*** **(%)**		
Moderate		18 (72.0)
Severe		7 (28.0)
**Asthma control status,** ***n*** **(%)**		
Controlled (ACQ score < 1.5 at screening)		16 (64.0)
Uncontrolled (ACQ score ≥ 1.5 at screening)		9 (36.0)
**FEV**_**1**_ **(L), mean (SD)**		2.2 (0.8)
**FEV**_**1**_**% predicted, mean (SD)**		62.1 (11.5)
**FEV**_**1**_**/FVC, mean (SD)**		0.7 (0.1)
**Number of patients experiencing exacerbations in the past 12 months,** ***n*** **(%)**		12 (48.0)
**Number of exacerbations in the last 12 months, mean (SD)**		1.1 (1.6)
Phase 2		
**Characteristics**	**205715 (*N*** **= 2270)**^**d**, **e**^	**205832 (*N*** **= 420)**^**e**^
**Age, years, mean (SD)**	53.3 (13.12)	48.8 (14.65)
**Females,** ***n*** **(%)**	1403 (61.8)	297 (70.7)
**Hispanic or Latino ethnicity,** ***n*** **(%)**	235 (10.4)	9 (2.1)
**Race,** ***n*** **(%)**		
White	1888 (83.2)	386 (91.9)
Asian	279 (12.3)	6 (1.4)
Black	84 (3.7)	26 (6.2)
Other	17 (0.7)	2 (0.5)
Missing	2 (0.1)	0 (0.0)
**FEV**_**1**_ **(L)**^f^**, mean (SD)**	2.0 (0.74)	2.2 (0.67)
**FEV**_**1**_**% predicted**^f^**, mean (SD)**	70.7 (14.29)	70.9 (11.13)
**Exacerbations in last 12 months, mean (SD)**	1.4 (1.29)	0.3 (0.72)
**Smoking history,** ***n*** **(%)**		
Never	1835 (80.8)	388 (92.4)
Former	430 (18.9)	32 (7.6)
Missing	5 (0.2)	0 (0.0)
**E-RS: COPD Total Score, mean (SD)**	8.3 (6.30)	7.2 (5.66)
**ACQ-5, mean (SD)**	2.0 (0.77)	1.8 (0.61)

*ACQ* Asthma Control Questionnaire, *FEV*_*1*_ Forced expiratory volume in 1 s, *FVC* Forced vital capacity, *ITT* Intent-to-treat, *SD* Standard deviation

^a^From self-reported quantitative patient data; ^b^not mutually exclusive; ^c^from site-reported quantitative data; ^d^interim blinded data only; ^e^2 ITT participants (205715) and 1 ITT participant (205832) with only 1 day or no pre-baseline data were excluded; ^f^based on pre-bronchodilator spirometry

#### Concept elicitation interviews

**Fig. 1:**
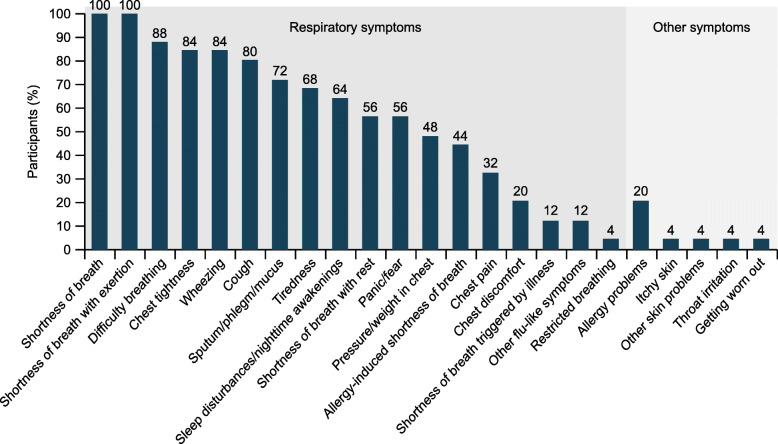
Proportion of patients reporting symptoms in the qualitative study (*N* = 25)

**Fig. 2:**
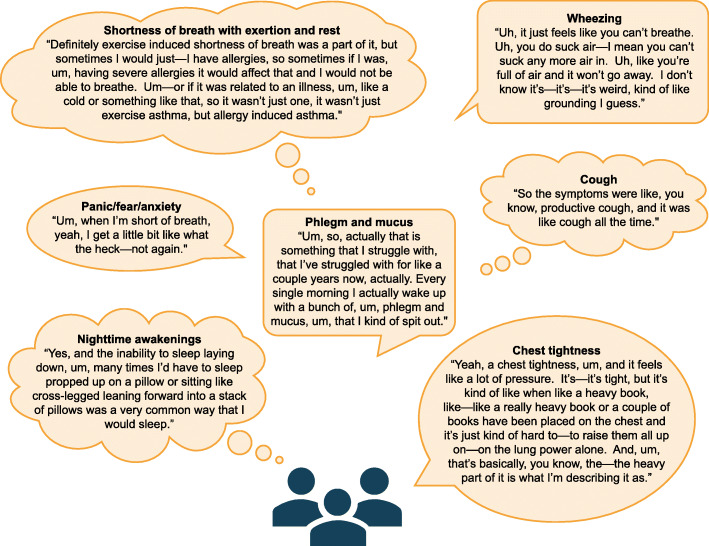
Patient core descriptions of respiratory symptoms captured during concept elicitation interviews

All patients reported multiple respiratory symptoms. Overall, 24 symptoms were described (Fig. 1), with 88% reported within the first seven interviews. Concept saturation was achieved by Interview 15. The most frequently reported symptoms included SOB with and without activity, difficulty breathing, and chest tightness (Fig. 1, Fig. 2).

#### Concept mapping

Mapping of concepts from concept elicitation interviews to items in the E-RS: COPD showed that they were relevant to patients with asthma, and patients’ descriptions were consistent with how symptoms are described in the E-RS: COPD. Symptoms in the E-RS: COPD were also largely consistent with those previously identified during a literature review and clinician interviews (Table 2).

**Table 2:** Item concepts mapped to E-RS: COPD and supplemental items in the qualitative study

E-RS: COPD items	Item concept reported in literature	Item concept reported by clinicians (***N*** = 2)	Item concept reported during patient interviews		
			Spontaneous	Probed	Total, *n* (%) (***N*** = 25)
Item 1: Chest congestion^a^	✓	✓	4	14	18 (72)
Item 2: Cough	✓	✓	8	12	20 (80)
Item 3: Mucus/phlegm^a^	✓	✓	4	14	18 (72)
Item 4: Difficulty bringing up mucus/phlegm^a^	✓	✓	4	14	18 (72)
Item 5: Chest discomfort	NR	NR	0	5	5 (20)
Item 6: Chest tightness	✓	✓	7	15	22 (88)
Item 7: Breathlessness	✓	✓	13	9	22 (88)
Item 8: Description of breathlessness	✓	✓	13	9	22 (88)
Item 9: Short of breath with personal care activities^b^	✓	✓	1	13	14 (56)
Item 10: Short of breath with indoor activities	✓	✓	17	8	25 (100)
Item 11: Short of breath with outdoor activities^c^	✓	✓	6	19	25 (100)

^a^Patients described various aspects of sputum/mucus production; ^b^patients described shortness of breath at rest or with minimal physical activity; ^c^patients described shortness of breath with exertion. ✓, reported; E-RS: COPD, Evaluating Respiratory Symptoms in Chronic Obstructive Pulmonary Disease; NR, not reported

#### Cognitive debriefing

All patients reported that instructions were clear and understandable. Overall, feedback was positive, and there were no reports of any difficulties completing the E-RS: COPD; 24/25 (96%) of patients indicated a clear understanding of the PRO (one patient was not asked). Most patients (19/25; 76%) reported that all items were applicable to their personal experience, and 21/25 (84%) thought the items were relevant to all patients with asthma. No key respiratory symptoms were missing.

### Phase 2: quantitative psychometric evaluation

A total of 421 patients were included in 205832 and 2270 patients were included in 205715. Most participants were female (205832: *n* = 297, 70.7%; 205715: *n* = 1403, 61.8%), with a mean (SD) age of 48.8 (14.7) years for the 205832 study, and 53.3 (13.1) years for the 205715 study (Table 1). As patients in the 205715 study were diagnosed with moderate/severe asthma, they had worse clinical characteristics compared with patients with moderate asthma in the 205832 study, highlighted by more exacerbations in the prior 12 months (1.4 vs 0.3), and higher baseline ACQ-5 (2.0 vs 1.8), and E-RS: COPD Total scores (8.3 vs 7.2).

#### Factor structure

CFA supported the fit of the data to the E-RS: COPD factor structure at baseline in 205832 (CFI = 0.872; SRMS = 0.061; RMSEA = 0.194) and in 205715 (CFI = 0.921; SRMR = 0.046; RMSEA = 0.154) (Supplementary Table 6). Findings supported retaining the E-RS: COPD factor structure for the 11 items for use in the moderate/severe asthma patient population. The inclusion of the two supplemental items (wheeze and SOB with strenuous physical activity) did not improve the model and were not included in the final scale. Therefore, the scoring algorithm from the E-RS: COPD was retained for subsequent analyses. The resulting tool is referred to as E-RS: Asthma in this context; subscale names are consistent across both tools.

#### Reliability

High Cronbach’s alpha coefficients (> 0.70) were observed for RS-Total score in 205715, ranging from 0.957 at screen run-in, 0.955 at Week 0, 0.954 at Week 24, and 0.951 at Week 52. Alpha coefficients were similarly high for RS subscale scores at all timepoints, including RS-Breathlessness (0.939–0.956), RS-Cough and Sputum (0.837–0.881), and RS-Chest Symptoms (0.935–0.950). Test–retest reliability data indicated stability of RS-Total and subscale scores in both trials, with ICC coefficient values > 0.70 for all assessment timepoints.

#### Validity

Acceptable construct validity was demonstrated. In 205715, at baseline (Week 0), moderate relationships (*r* > 0.40) were observed between RS-Total score and SGRQ-Total, SGRQ Symptoms and SGRQ Activity domains (range 0.44–0.48, all *P* < 0.0001). Similar relationships were observed at Week 24, but correlations were higher (range 0.51–0.55, all *P* < 0.0001). RS-Total and subscale scores showed evidence of convergent validity with asthma symptom severity in both studies, with high correlations at baseline (0.64–0.88) and at Week 24 (0.65–0.86, all *P* < 0.0001). In support of known-groups validity, when PGI-S was used as an anchor, mean scores on the E-RS: Asthma increased linearly with increasing global severity in both trials (Table 3).

**Table 3:** Known-groups validity of mean weekly RS-total and subscales at baseline in 205715/205832

Patient groups	205715^a^				205832			
	Total (*n*, mean [SD])	Breathlessness (*n*, mean [SD])	Cough and sputum (*n*, mean [SD])	Chest symptoms (*n*, mean (SD))	Total (*n*, mean [SD])	Breathlessness (*n*, mean [SD])	Cough and sputum (*n*, mean [SD])	Chest symptoms (*n*, mean [SD])
**Exacerbation history (number)**								
≥ 2	638, 9.19 (0.25)	638, 4.44 (0.13)	638, 2.40 (0.07)	638, 2.35 (0.07)	26, 8.46 (1.11)	26, 3.95 (0.59)	26, 2.28 (0.30)	26, 2.23 (0.34)
1	1284, 7.86 (0.18)	1284, 3.76 (0.09)	1284, 2.13 (0.05)	1284, 1.97 (0.05)	71, 7.41 (0.67)	71, 3.42 (0.36)	71, 2.11 (0.18)	71, 1.88 (0.20)
0	317, 8.15 (0.35)	317, 3.85 (0.18)	317, 2.19 (0.10)	317, 2.11 (0.10)	323, 7.06 (0.31)	323, 3.43 (0.17)	323, 1.82 (0.09)	323, 1.80 (0.10)
F-test statistic	9.64	9.38	5.23	8.87	0.80	0.36	1.84	0.79
*P*-value	< 0.0001	< 0.0001	0.0054	0.0001	0.4497	0.6961	0.1608	0.4551
**FEV**_**1**_**% predicted**								
Mild (≥80%)	448, 6.59 (0.29)	448, 3.00 (0.15)	448, 1.91 (0.08)	448, 1.67 (0.09)	102, 7.06 (0.56)	102, 3.19 (0.30)	102, 1.98 (0.15)	102, 1.88 (0.17)
Moderate (50–< 80%)	1469, 8.34 (0.16)	1469, 4.02 (0.08)	1469, 2.20 (0.05)	1469, 2.12 (0.05)	298, 7.09 (0.33)	298, 3.47 (0.17)	298, 1.82 (0.09)	298, 1.80 (0.10)
Severe (30–< 50%)	282, 10.64 (0.37)	282, 5.17 (0.19)	282, 2.79 (0.10)	282, 2.68 (0.11)	19, 10.07 (1.29)	19, 4.92 (0.68)	19, 2.77 (0.35)	19, 2.38 (0.39)
Very severe (< 30%)	9, 13.26 (2.06)	9, 6.83 (1.07)	9, 3.34 (0.58)	9, 3.09 (0.62)	0, N/A	0, N/A	0, N/A	0, N/A
F-test statistic	26.85	29.03	16.16	18.41	2.55	2.71	3.54	1.07
*P*-value	< 0.0001	< 0.0001	< 0.0001	< 0.0001	0.0794	0.0678	0.0300	0.3429
**PGI-S**								
None	52, 1.87 (0.81)	52, 0.81 (0.42)	52, 0.67 (0.23)	52, 0.40 (0.24)	5, 3.40 (2.38)	5, 1.07 (1.27)	5, 1.38 (0.67)	5, 0.96 (0.73)
Mild	687, 5.36 (0.22)	687, 2.46 (0.12)	687, 1.53 (0.06)	687, 1.37 (0.07)	197, 5.34 (0.38)	197, 2.53 (0.20)	197, 1.48 (0.11)	197, 1.33 (0.12)
Moderate	1384, 9.62 (0.16)	1384, 4.64 (0.08)	1384, 2.55 (0.04)	1384, 2.43 (0.05)	213, 8.84 (0.37)	213, 4.29 (0.19)	213, 2.27 (0.10)	213, 2.28 (0.11)
Severe	97, 13.39 (0.59)	97, 6.58 (0.31)	97, 3.26 (0.17)	97, 3.55 (0.18)	5, 14.47 (2.38)	5, 7.11 (1.27)	5, 2.98 (0.67)	5, 4.38 (0.73)
Very severe	1, 22.60 (5.82)	1, 10.00 (3.04)	1, 6.20 (1.65)	1, 6.40 (1.77)	0, N/A	0, N/A	0, N/A	0, N/A
F-test statistic	97.27	91.94	66.37	70.99	18.66	17.16	10.48	16.19
*P*-value	< 0.0001	< 0.0001	< 0.0001	< 0.0001	< 0.0001	< 0.0001	< 0.0001	< 0.0001

^a^Interim blinded data only

*FEV*_*1*_ Forced expiratory volume in 1 s, *N/A* Not applicable, *PGI-S* Patient Global Impression of Severity, *SD* Standard deviation

#### Responsiveness

**Fig. 3:**
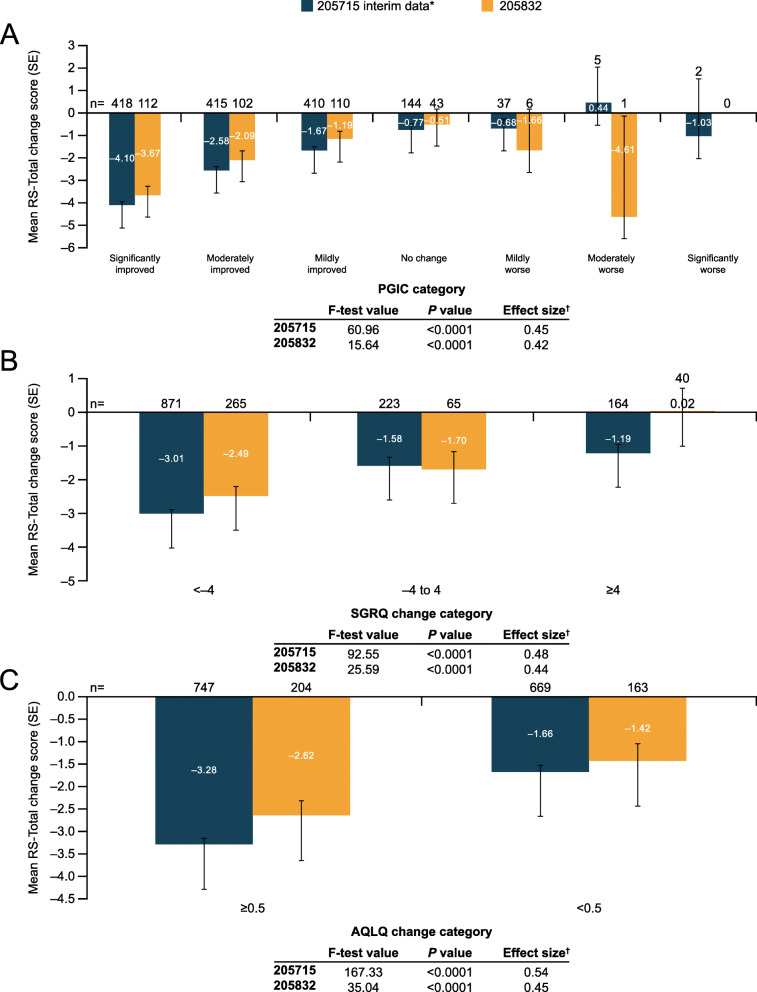
Responsiveness of weekly RS-Total scores using **A** PGIC, **B** SGRQ, and **C** AQLQ. *Note:* ANCOVA of E-RS change scores from baseline (Week 0) to Week 24 in 205715 and 205832 studies. *Interim blinded data only; ^†^Among patients defined as “responders” (PGIC improvement, SGRQ change <− 4, or AQLQ change ≥0.5). AQLQ, Asthma Quality of Life Questionnaire; PGIC, Patient Global Impression of Change; SE, standard error; SGRQ, St George’s Respiratory Questionnaire

When PGIC was used as an anchor, statistically significant differences were observed between least squares (LS) mean RS-Total and subscale scores from baseline to Week 4, baseline to Week 12, and baseline to Week 24 (all *P* < 0.0001) (Fig. 3A). Observed effect sizes were moderate at all timepoints (205832: 0.42; 205715: 0.45). When SGRQ was used as an anchor, LS mean RS-Total and subscale scores showed the greatest improvement in the SGRQ change group of <− 4 compared with the other two SGRQ groups, from baseline to Weeks 4, 12, and 24 (all *P* < 0.0001) (Fig. 3B), with moderate effect sizes observed at all timepoints (205832: 0.44; 205715: 0.48). Lastly, when AQLQ was used as an anchor, patients with AQLQ change ≥0.5 between baseline and Weeks 4, 12, and 24 also showed the greatest reduction in E-RS: Asthma scores (all *P* < 0.0001) (Fig. 3C), with moderate effect sizes observed at all timepoints (205832: 0.45; 205715: 0.54).

#### Responder threshold

Among patients “mildly improved” on the PGIC, mean (SD) changes of − 1.5 (5.70) and − 1.9 (3.46) were seen for RS-Total score in 205832 and 205715, with respective changes of − 0.8 (2.84) and − 0.9 (1.72), − 0.3 (1.76) and − 0.5 (1.27), and − 0.4 (1.73) and − 0.5 (1.18) for the RS-Breathlessness, RS-Cough and Sputum, and RS-Chest Symptom scores. CDFs are presented in the online supplement (Supplementary Figure 1). Similar changes in RS-Total and subscales were observed when SQRQ and AQLQ were used as anchors. Thus, among patients with a reduction of ≥4 on the SGRQ, mean (SD) changes of − 2.4 (4.74) and − 3.0 (4.31) were seen for RS-Total score in 205832 and 205715, with respective changes of − 1.1 (2.35) and − 1.4 (2.14), − 0.7 (1.59) and − 0.8 (1.39), and − 0.6 (1.45) and − 0.8 (1.39) for the RS-Breathlessness, RS-Cough and Sputum, and RS-Chest Symptom scores. Among patients with a change of ≥0.5 on the AQLQ, mean (SD) changes of − 2.6 (5.11) and − 3.4 (4.46) were seen for RS-Total score in 205832 and 205715, with respective changes of − 1.2 (2.59) and − 1.6 (2.23), − 0.7 (1.58) and − 0.9 (1.45), and − 0.7 (1.52) and − 1.0 (1.45) for the RS-Breathlessness, RS-Cough and Sputum, and RS-Chest Symptom scores. The results of the distribution-based approach for score interpretation, using SEM and half SD values for the RS-Total score ranged from 2.8 to 3.6, with lower scores found for the subscale scores ranging from 1.5 to 1.9 for RS-Breathlessness, 0.8 to 1.1 for RS-Cough and Sputum, and 0.9 to 1.1 for RS-Chest Symptoms (Supplementary Table 7).

Based on the totality of evidence across both 205832 and 205715, the proposed meaningful change thresholds for the E-RS: Asthma are as follows: RS-Total, − 2.0; RS-Breathlessness, − 1.0; RS-Cough and Sputum, − 0.7; and RS-Chest Symptoms, − 0.7.

## Discussion

The EXACT and E-RS: COPD were the first PROs to be qualified by the US FDA under the drug development tools initiative for use in COPD [32]. While these were validated as a daily diary in COPD, their content validity and performance in patients with moderate/severe asthma had not been assessed. A literature review highlighted that the Asthma Daytime Symptom Diary was a potential alternative candidate PRO to assess asthma symptoms, but was at an earlier stage of development [33].

The FDA has indicated a willingness to consider modified or adapted PROs for use in related disease areas [34], and earlier studies have shown the E-RS: COPD to be reliable, valid and responsive in a sample of patients with ACO [24]. Therefore, we hypothesized that the E-RS: COPD could be a suitable candidate for use in patients with asthma due to the overlap in symptoms reported in the literature and the use of other tools for the periodic assessment of asthma and COPD [7–9].

In this report, a two-phase qualitative and quantitative analysis was used to evaluate whether the E-RS: COPD was suitable for assessing respiratory symptoms in patients with moderate/severe asthma. In Phase 1, qualitative interviews with 25 patients with moderate/severe asthma were conducted. These interviews established content validity; the instrument captured the most relevant and important respiratory symptoms of asthma. Additionally, the items, response options, and recall period were understood, acceptable, and appropriate in this patient population. In Phase 2, the psychometric properties of the tool were evaluated using data from two separate clinical trials (205832 [11] and 205715 [12]). The findings indicated that the fit of data to the E-RS: COPD factor structure was acceptable and, the same scoring algorithm from the E-RS: COPD was retained for use for all subsequent psychometric analyses in the asthma population (and referred to as E-RS: Asthma). The E-RS: Asthma demonstrated good psychometric properties and was highly responsive in patients with moderate/severe asthma. The proposed responder threshold of ≥ − 2.0 points on the RS-Total score can be used by clinicians to better understand and interpret clinical trial results and may identify responders to treatment.

Although wheeze is a concept that is consistently reported by patients with asthma, it is inconsistently described, challenging to translate, and is interpreted in different ways by patients in different countries. Due to this, and requirements for linguistic validation in different languages, previous studies found that a wheeze item did not load with the 11 items in the E-RS when assessed in COPD or ACO populations [6, 24]. Given differences in the frequency and severity of symptoms in patients with asthma compared to those with COPD, an additional item related to SOB with more strenuous activity was also tested since there is a possibility that symptoms and their impacts can be reversed in asthma such that more strenuous activity is possible. However, this item also did not load with the E-RS items when evaluated in a population with moderate/severe asthma and was also highly correlated with the “SOB on outdoor activities” item in the E-RS: COPD. It was therefore deemed to be redundant. Therefore, the findings from the EFA in relation to the supplemental items included in our analysis were anticipated.

Some limitations should be considered in the interpretation of our findings, primarily in relation to generalizability. First, the mean (SD) age of study populations (qualitative cross-sectional study: 48 ± 16.5 years; 205832: 48.8 ± 14.7 years; 205715: 53.3 ± 13.1 years) may limit the generalizability of the findings to younger patients, particularly adolescents where further qualitative research may be required. However, it should be noted that there was no upper age limit for enrollment in both trials, indicating that our findings are potentially relevant to a broad age range of adults. Second, patients in both phases of the analysis came from clinical studies that studied either moderate or moderate/severe asthma, thus our findings may not be applicable to patients with milder disease. Finally, in the qualitative study, patients were based in the USA, and were predominantly Caucasian, so findings may not be applicable to other countries, cultures, and ethnicities. Whilst Spanish-speaking patients were eligible and substantial effort was made to recruit Spanish-speaking participants, only one such person was interviewed. Nevertheless, the sample size (*N* = 25) was reasonably large for a qualitative study, and was sufficient to reach saturation; however, a more diverse sample may have captured additional concepts. Additionally, no conclusions regarding use of the tool in Spanish-speaking asthma populations can be drawn on the basis of this study, although substantial testing of the Spanish EXACT translation (14 items which include the E-RS: COPD) has been completed amongst native Spanish-speakers with COPD in the US, Spain, Mexico, Chile, Argentina, Peru, Colombia and Guatemala, indicating that the respiratory terms used are likely applicable to a broad range of Spanish speakers with respiratory illness.

There are a number of strengths to this study. First, we used a step-wise approach to evaluate content validity of an existing measure (E-RS: COPD) for use in a new disease, asthma, in line with FDA guidance [34] and the International Society of Pharmacoeconomics and Outcomes Research (ISPOR) guidelines [35]. Additionally, the psychometric evaluation study demonstrated support for the psychometric properties of the E-RS: Asthma in two large clinical trial populations with moderate/severe asthma. Similar results were obtained across the two separate studies, providing robust evidence of the properties of the instrument in this patient population and making it applicable to wider use in evaluating asthma patients and their response to treatment.

## Conclusions

The E-RS: COPD was found to be valid and reliable for use in this asthma population. The tool will be referred to as E-RS: Asthma when used in asthma populations. The instrument was found to be comprehensive, relevant, acceptable, and understood by patients with moderate/severe asthma. Psychometric analyses support the use of the E-RS: COPD scoring structure in this patient population and the E-RS: Asthma tool showed reliability, validity, and responsiveness, in patients with moderate/severe asthma.

## Supplementary Information

**Additional file 1: Figure S1.** Cumulative distribution function of RS-Total Change score by PGIC in (A) 205832 and (B) 205715 studies. **Table S1.** Eligibility criteria for the qualitative study (*N* = 25). **Table S2.** Eligibility criteria for GSK 205832 included in the psychometric evaluation. **Table S3.** Eligibility criteria for GSK 205715 for inclusion in the psychometric evaluation. **Table S4.** Study visit schedule for PRO collection in 205832 and 205715. **Table S5.** Summary of clinical trials 205832 and 205715. **Table S6.** Confirmatory factor analysis of E-RS: COPD at Week 0 in 205832 and 205715 studies. **Table S7.** Distribution-based approaches for the E-RS: Asthma.

## Data Availability

Anonymized individual participant data and study documents can be requested for further research from www.clinicalstudydatarequest.com.

## Abbreviations

ACO: Asthma–COPD overlap
ACQ: Asthma Control Questionnaire
ADSD: Asthma Daytime Symptom Diary
ANCOVA: Analysis of covariance
ANOVA: Analysis of variance
AQLQ: Asthma Quality of Life Questionnaire
CDF: Cumulative distribution function
CFA: Confirmatory factor analysis
CFI: Comparative fit index
COPD: Chronic obstructive pulmonary disease
COREQ: Consolidated Criteria for Reporting Qualitative Research
DPI: Dry powder inhaler
eDiary: Electronic diary
EFA: Exploratory factor analysis
E-RS: Asthma: Evaluating Respiratory Symptoms in Asthma
E-RS: COPD: Evaluating Respiratory Symptoms in Chronic Obstructive Pulmonary Disease
EXACT: EXAcerbations of Chronic Pulmonary Disease Tool
FDA: Food and Drug Administration
FEV_1_: Forced expiratory volume in 1 s
FF: Fluticasone furoate
FP: Fluticasone propionate
FVC: Forced vital capacity
ICC: Intra-class correlation
ICS: Inhaled corticosteroids
IRB: Institutional Review Board
ISPOR: International Society of Pharmacoeconomics and Outcomes Research
ITT: Intent-to-treat
LABA: Long-acting β_2_-agonist
LAMA: Long-acting muscarinic antagonist
LS: Least squares
NR: Not reported
PGIC: Patient Global Impression of Change
PGI-S: Patient Global Impression of Severity
PRO: Patient-reported outcome
QD: Once daily
RCT: Randomized controlled trial
RMSEA: Root mean square error of approximation
SABA: Short-acting β_2_-agonist
SE: Standard error
SEM: Standard error of measurement
SD: Standard deviation
SGRQ: St George’s Respiratory Questionnaire
SOB: Shortness of breath
SRMR: Standardized root mean residual
UMEC: Umeclidinium bromide
VI: Vilanterol

## References

[1] Global Initiative for Asthma (2020) Global strategy for asthma management and prevention. https://ginasthma.org/. Accessed May 2020.

[2] Juniper EF (2005). Assessing asthma quality of life: Its role in clinical practice. Breathe.

[3] Juniper EF, Guyatt GH, Epstein RS, Ferrie PJ, Jaeschke R, Hiller TK (1992). Evaluation of impairment of health related quality of life in asthma: Development of a questionnaire for use in clinical trials. Thorax.

[4] Pereira ED, Cavalcante AG, Pereira EN, Lucas P, Holanda MA (2011). Asthma control and quality of life in patients with moderate or severe asthma. Jornal Brasileiro de Pneumologia.

[5] Leidy NK, Wilcox TK, Jones PW, Roberts L, Powers JH, Sethi S (2011). Standardizing measurement of chronic obstructive pulmonary disease exacerbations. Reliability and validity of a patient-reported diary. American Journal of Respiratory and Critical Care Medicine.

[6] Leidy NK, Murray LT, Monz BU (2014). Measuring respiratory symptoms of COPD: Performance of the EXACT- respiratory symptoms tool (E-RS) in three clinical trials. Respiratory Research.

[7] Buist AS, DJ BP, Rennard S (2001). Definitions. *Asthma and COPD textbook; basic mechanisms and clinical management*.

[8] Buist AS (2003). Similarities and differences between asthma and chronic obstructive pulmonary disease: Treatment and early outcomes. European Respiratory Journal.

[9] Nelsen L, Gater A, Panter C, Tolley C, Lee L, Pascoe S (2017). Understanding and measuring symptoms and health status in asthma COPD overlap: Content validity of the EXACT and SGRQ. Journal of Patient-Reported Outcomes.

[10] Global Initiative for Chronic Obstructive Lung Disease (2021) Global strategy for the diagnosis, management and prevention of chronic obstructive pulmonary disease. https://goldcopd.org/wp-content/uploads/2020/11/GOLD-REPORT-2021-v1.1-25Nov20_WMV.pdf. Accessed 4 February 2021.

[11] Kerwin E, Pascoe S, Bailes Z (2020). A phase IIb, randomised, parallel-group study: The efficacy, safety and tolerability of once-daily umeclidinium in patients with asthma receiving inhaled corticosteroids. Respiratory Research.

[12] Lee LA, Bailes Z, Barnes N, Boulet LP, Edwards D, Fowler A (2021). Efficacy and safety of once-daily single-inhaler triple therapy (FF/UMEC/VI) versus FF/VI in patients with inadequately controlled asthma (CAPTAIN): A double-blind, randomised, phase 3A trial. The Lancet Respiratory Medicine.

[13] Leidy NK, Vernon M (2008). Perspectives on patient-reported outcomes : Content validity and qualitative research in a changing clinical trial environment. Pharmacoeconomics.

[14] Tong A, Sainsbury P, Craig J (2007). Consolidated criteria for reporting qualitative research (COREQ): A 32-item checklist for interviews and focus groups. International Journal for Quality in Health Care.

[15] Khusial RJ, Honkoop PJ, van der Meer V, Snoeck-Stroband JB, Sont JK (2020). Validation of online asthma control questionnaire and asthma quality of life questionnaire. ERJ Open Research.

[16] US Food and Drug Administration (FDA) (2018) Chronic obstructive pulmonary disease: Use of the St. George’s Respiratory Questionnaire as a PRO assessment tool guidance for industry. https://www.fda.gov/files/drugs/published/Chronic-Obstructive-Pulmonary-Disease%2D%2DUse-of-the-St.-George%E2%80%99s-Respiratory-Questionnaire-as-a-PRO-Assessment-Tool-Guidance-for-Industry.pdf. Accessed 16 May 2021.

[17] Leidy NK, Sexton CC, Jones PW, Notte SM, Monz BU, Nelsen L (2014). Measuring respiratory symptoms in clinical trials of COPD: Reliability and validity of a daily diary. Thorax.

[18] Jones PW, Quirk FH, Baveystock CM (1991). The St George's respiratory questionnaire. Respiratory Medicine.

[19] Jones PW, Quirk FH, Baveystock CM, Littlejohns P (1992). A self-complete measure of health status for chronic airflow limitation. The St. George's respiratory questionnaire. American Review of Respiratory Disease.

[20] Juniper EF, Guyatt GH, Willan A, Griffith LE (1994). Determining a minimal important change in a disease-specific quality of life questionnaire. Journal of Clinical Epidemiology.

[21] Juniper EF, O'Byrne PM, Guyatt GH, Ferrie PJ, King DR (1999). Development and validation of a questionnaire to measure asthma control. European Respiratory Journal.

[22] Juniper, E. F., Bousquet, J., Abetz, L., & Bateman, E. D. (2006). Identifying 'well-controlled' and 'not well-controlled' asthma using the asthma control questionnaire. *Respiratory Medicine*, *100*(4), 616–621. 10.1016/j.rmed.2005.08.012. 10.1016/j.rmed.2005.08.012 16226443

[23] Juniper EF, Svensson K, Mörk AC, Ståhl E (2005). Measurement properties and interpretation of three shortened versions of the asthma control questionnaire. Respiratory Medicine.

[24] Nelsen LM, Lee LA, Wu W, Lin X, Murray L, Pascoe SJ, Leidy NK (2019). Reliability, validity and responsiveness of E-RS:COPD in patients with spirometric asthma-COPD overlap. Respiratory Research.

[25] Byrne B (2011). *Structural equation modeling with Mplus: Basic concepts, applications, and programming*.

[26] Kline RB (2005). *Principles and practice of structural equation modeling*.

[27] Browne M, Cudeck R, Bollen KA, Long JS (1993). Alternative ways of assessing model fit. *Testing structural equation models*.

[28] Koo TK, Li MY (2016). A guideline of selecting and reporting Intraclass correlation coefficients for reliability research. Journal of Chiropractic Medicine.

[29] Wyrwich KW, Tierney WM, Wolinsky FD (1999). Further evidence supporting an SEM-based criterion for identifying meaningful intra-individual changes in health-related quality of life. Journal of Clinical Epidemiology.

[30] Wyrwich KW, Nienaber NA, Tierney WM, Wolinsky FD (1999). Linking clinical relevance and statistical significance in evaluating intra-individual changes in health-related quality of life. Medicine Care.

[31] Norman GR, Sloan JA, Wyrwich KW (2003). Interpretation of changes in health-related quality of life: The remarkable universality of half a standard deviation. Medicine Care.

[32] Leidy NK, Murray LT (2013). Patient-reported outcome (PRO) measures for clinical trials of COPD: The EXACT and E-RS. COPD.

[33] Gater A, Nelsen L, Fleming S, Lundy JJ, Bonner N, Hall R, Patient-Reported Outcome Consortium’s Asthma Working Group (2016). Assessing asthma symptoms in adolescents and adults: Qualitative research supporting development of the asthma daily symptom diary. Value Health.

[34] US Food and Drug Administration (FDA) (2018) Patient-focused drug development guidance: Methods to identify what is important to patients and select, develop or modify fit-for-purpose clinical outcome assessments. https://www.fda.gov/drugs/news-events-human-drugs/patient-focused-drug-development-guidance-methods-identify-what-important-patients-and-select. Accessed 28 April 2020.

[35] Rothman M, Burke L, Erickson P, Leidy NK, Patrick DL, Petrie CD (2009). Use of existing patient-reported outcome (PRO) instruments and their modification: The ISPOR good research practices for evaluating and documenting content validity for the use of existing instruments and their modification PRO task force report. Value Health.

