# Supplementary material for: Measuring respiratory symptoms in moderate/severe asthma: evaluation of a respiratory symptom tool, the E-RS®: COPD in asthma populations
Source: J Patient Rep Outcomes. 2021 Oct 10;5:104. doi: 10.1186/s41687-021-00338-6 (PMC8502721; doi:10.1186/s41687-021-00338-6)
Supplement: Supplementary file 1 — Additional file 1: Figure S1. Cumulative distribution function of RS-Total Change score by PGIC in (A) 205832 and (B) 205715 studies. Table S1. Eligibility criteria for the qualitative study (N = 25). Table S2. Eligibility criteria for GSK 205832 included in the psychometric evaluation. Table S3. Eligibility criteria for GSK 205715 for inclusion in the psychometric evaluation. Table S4. Study visit schedule for PRO collection in 205832 and 205715. Table S5. Summary of clinical trials 205832 and 205715. Table S6. Confirmatory factor analysis of E-RS: COPD at Week 0 in 205832 and 205715 studies. Table S7. Distribution-based approaches for the E-RS: Asthma. [file 41687_2021_338_MOESM1_ESM.docx]

# Supplementary materials

## Figure Legends

**Supplementary Figure 1.** Cumulative distribution function of RS-Total Change score by PGIC in (A) 205832 and (B) 205715 studies


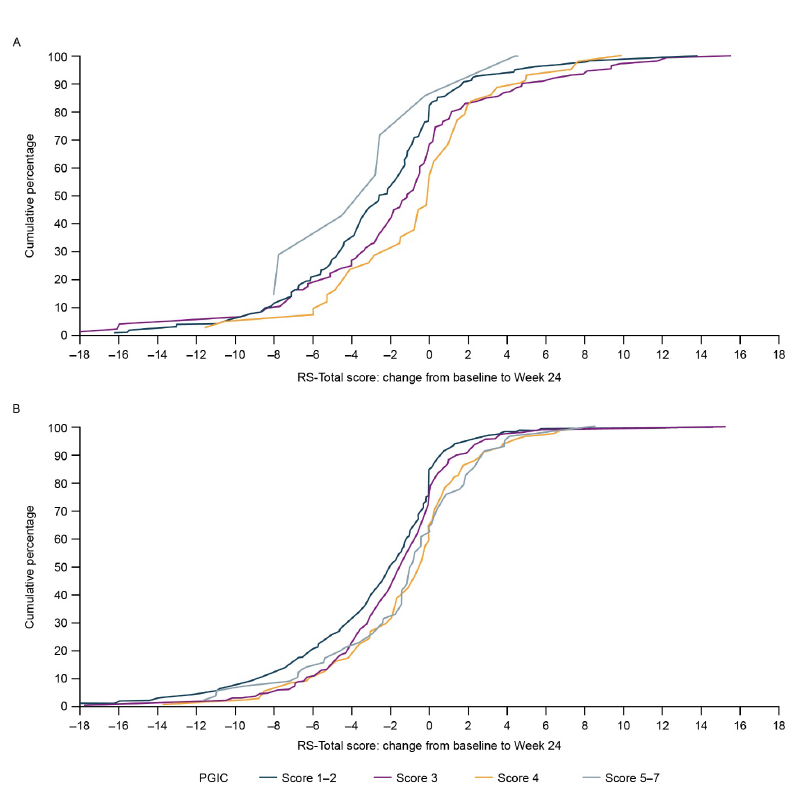


PGIC, Patient Global Impression of Change

## **Supplementary Table 1.** Eligibility criteria for the qualitative study (N=25)

| **Inclusion criteria** | **Exclusion criteria** |
| --- | --- |
| - ≥18 years at the time of consent | - Current smoker |
| - Pre-bronchodilator FEV₁ <80% predicted recorded in previous 12 months | - Emphysema or chronic bronchitis (COPD other than asthma) |
| - Post-bronchodilator increase in FEV_1_ of ≥12% and ≥200 mL in the previous 12 months - Moderate/severe asthma as defined by a stable ICS dose for ≥12 weeks prior to screening   - Moderate: ICS dose >250–500 mcg/day FP (or equivalent)   - Severe: ICS dose >500 mcg/day FP (or equivalent) | - Clinically important lung condition other than asthma (eg, current infection, bronchiectasis, pulmonary fibrosis, bronchopulmonary aspergillosis, eosinophilic disorder such as eosinophilic esophagitis, history of lung cancer) - Participated in an interventional study within the past 30 days |
| - Documented control status using ACQ-6* items at screening |  |
| - Able to understand, read, and speak English or Spanish sufficiently to complete all assessments |  |
| - Willing and able to take part in a telephone interview session |  |
| - Willing and able to provide written informed consent |  |

*ACQ-6 comprises five questions on symptoms and impacts of asthma (ACQ-5) plus rescue use.
ACQ, asthma control questionnaire; COPD, chronic obstructive pulmonary disease; FEV_1_, forced expiratory volume in 1 second; FP, fluticasone propionate; ICS, inhaled corticosteroid

## **Supplementary Table 2.** Eligibility criteria for GSK 205832 included in the psychometric evaluation

|  | **Inclusion criteria** | **Exclusion criteria** |
| --- | --- | --- |
| **Eligibility criteria at screening** | - Male or female ≥18 years of age. Women of reproductive potential must be using effective contraception | - Women who are pregnant, lactating or who are planning on becoming pregnant |
|  | - Written informed consent | - Patients at risk of non-compliance, or unable to comply with the study procedures; and patients with conditions that would affect validity of consent |
|  | - Asthma diagnosis for ≥6 months (as defined by the NIH [1]) at  Visit 0 | - Chest X-ray-confirmed pneumonia in the 12 weeks prior to Visit 1 or risk factors for pneumonia |
|  | - Partially controlled or uncontrolled asthma (ACQ-6* ≥0.75 at  Visit 1 | - Severe asthma exacerbation within 12 weeks of Visit 1 |
|  | - Receiving continuous ICS (FP ≥100 mcg/day or equivalent) ± LABA or LAMA for ≥12 weeks, with no change in asthma therapy for the previous 4 weeks and able to withhold rescue medication for ≥6 hours before each visit | - Concurrent respiratory disease other than asthma such as pneumonia, pneumothorax, atelectasis, pulmonary fibrotic disease, bronchopulmonary dysplasia, chronic bronchitis, emphysema, COPD, lung cancer |
|  | - Best pre-bronchodilator morning FEV_1_ ≤90% of the predicted normal AND best post-bronchodilator FEV_1_/FVC ≥0.7 at Visit 1 | - Unstable liver disease |
|  | - Airway reversibility, defined as an increase of ≥12% and ≥200 mL in FEV_1_ 20–60 minutes following four inhalations of salbutamol at Visit 1^†^ | - Unstable or life-threatening cardiac disease |
|  | - Ability to replace current SABA inhaler with salbutamol at Visit 1 for the study duration | - Clinically significant ECG abnormality |
|  |  | - Cancer |
|  |  | - Other disease or abnormality that could compromise patient safety or affect the analysis |
|  |  | - Medical conditions potentially impacted by a muscarinic receptor antagonist  (eg, narrow angle glaucoma, urinary retention, prostatic hypertrophy, bladder neck obstruction)^‡^ |
|  |  | - Unable to withhold salbutamol for 6 hours prior to spirometry |
|  |  | - Current smokers and former smokers (≥10 pack-years) and inhaled tobacco use within previous 12 months |
|  |  | - Drug or alcohol abuse |
|  |  | - Allergy or hypersensitivity to any corticosteroid, anticholinergic/muscarinic receptor antagonist or β_2_-agonist, lactose/milk protein or magnesium stearate |
| **Randomization criteria** | - ACQ-6 total score >0.75 at Visit 2 - Pre-bronchodilator morning FEV_1_ ≤90% predicted normal value at Visit 2 - ALT ≤2x ULN, ALP ≤1.5x ULN, bilirubin ≤1.5x ULN at Visit 1 - Compliance with completion of the eDiary (completion of all questions/assessments on ≥4/7 final days of the run-in period) | - Respiratory infection during the run-in period that led to change in asthma management or expected to affect asthma status or ability participate in the study - Moderate/severe asthma exacerbation during screening or run-in period - Change in asthma medication (excluding changes after Visit 0 or run-in medication and SABA provided at Visit 1) - Clinically significant abnormal laboratory tests during screening or run-in, which are still abnormal upon repeat analysis |

*ACQ-6 comprises five questions on symptoms and impacts of asthma (ACQ-5) plus rescue use; ^†^if the patient does not meet the reversibility criteria at Visit 1, the assessment may be repeated once within 7 days of Visit 1 if there is a ≥9% increase in FEV_1_ 20−60 minutes following four salbutamol inhalations or if there is documented evidence of a reversibility assessment within 1 year prior to Visit 1, which demonstrated a post-bronchodilator increase in FEV_1_ of ≥12% and ≥200 mL; ^‡^patients could be included at the discretion of the Investigator if the benefit of antimuscarinic therapy outweighs the risk and the condition would not contraindicate study participation.
ACQ, Asthma Control Questionnaire; ALT, alanine aminotransferase; ALP, alkaline phosphatase; COPD, chronic obstructive pulmonary disease; ECG, electrocardiogram; eDiary, electronic diary; FEV_1_, forced expiratory volume in 1 second; FVC, forced vital capacity; ICS, inhaled corticosteroid; LABA, long-acting β_2_-agonist; LAMA, long-acting muscarinic antagonist; NIH, National Institute of Health; SABA, short-acting β_2_-agonist; ULN, upper limit of normal

## **Supplementary Table 3.** Eligibility criteria for GSK 205715 for inclusion in the psychometric evaluation

|  | **Inclusion criteria** | **Exclusion criteria** |
| --- | --- | --- |
| **Eligibility criteria (screening/run-in)** | - Male or female ≥18 years of age. Women of reproductive potential must be using effective contraception | - Women who are pregnant, lactating, or who are planning on becoming pregnant |
|  | - Written informed consent | - Patients at risk of non-compliance, or unable to comply with the study procedures |
|  | - Asthma diagnosis for ≥1 year at Visit 0 | - Chest X-ray confirmed pneumonia in the 6 weeks prior to Visit 1 or risk factors for pneumonia |
|  | - Symptomatic, uncontrolled asthma (ACQ-6* score ≥1.5) despite ICS/LABA maintenance therapy at Visit 1 | - Asthma exacerbation in the 6 weeks prior to Visit 1^‡^ |
|  | - In the year prior to Visit 1, a documented healthcare visit for acute asthma symptoms or a documented temporary (non-permanent) change in asthma therapy for acute asthma symptoms (according to a pre-specified asthma action plan) | - COPD, defined by all three of the following (as per GOLD guidelines [2]): i) history of exposure to risk factors; ii) post-salbutamol FEV_1_/FVC ratio <0.70 and post-salbutamol FEV_1_ ≤70% of predicted normal value; iii) onset of disease after age 40 years |
|  | - Permitted maintenance medications included stable maintenance doses of biologics (eg, anti-immunoglobulin E and anti-interleukin-5 agents) provided that treatment was initiated ≥16 weeks prior to Visit 1), and systemic corticosteroids (≤5 mg/day prednisone [or equivalent dose of an alternative systemic corticosteroid]), provided that treatment was initiated ≥12 weeks prior to Visit 1 and was stable for the 8 weeks prior to Visit 1 | - Concurrent respiratory disorders (evidence of pneumonia, tuberculosis, lung cancer, significant bronchiectasis, sarcoidosis, lung fibrosis, pulmonary hypertension, interstitial lung disease or any active pulmonary disease or abnormality other than asthma) - Unstable liver disease |
|  | - The need for daily ICS/LABA for ≥12 weeks prior to Visit 0, with no changes to maintenance asthma medications during the 6 weeks immediately prior to Visit 0 (including no changes to a stable total dose of ICS of FP >250 mcg/day or equivalent) | - Unstable or life-threatening cardiac disease |
|  | - A best pre-bronchodilator morning FEV_1_ ≥30% and <85% of the predicted normal [3] value at Visit 1 | - Clinically significant ECG abnormality |
|  | - Airway reversibility, defined as an increase of ≥12% and ≥200 mL in FEV_1_ 20–60 minutes following four inhalations of salbutamol at Visit 1^†^ | - Cancer |
|  | - Ability to replace current SABA inhaler with salbutamol at Visit 1 for the study duration | - Other disease or abnormality that could compromise patient safety or affect the analysis |
|  |  | - Medical conditions potentially impacted by a muscarinic receptor antagonist  (eg narrow angle glaucoma, urinary retention, prostatic hypertrophy, bladder neck obstruction)^§^ |
|  |  | - Unable to withhold salbutamol for 6 hours prior to spirometry |
|  |  | - Current smokers (inhaled tobacco product use within 1 year prior to Visit 1 or former smokers (≥10 pack-years) |
|  |  | - Drug or alcohol abuse |
|  |  | - Allergy or hypersensitivity to any corticosteroid, anticholinergic/muscarinic receptor antagonist or β_2_-agonist, lactose/milk protein or magnesium stearate - Patients with conditions that would affect validity of consent |
| **Enrollment criteria (enrollment/stabilization)** | - Inadequately controlled asthma (ACQ-6 score ≥1.5) at  Visit 2 | - Respiratory infection during the run-in period expected to affect asthma status or ability participate in the study |
|  | - A best pre-bronchodilator morning FEV_1_ ≥30% and <90% of the predicted normal [3] value at Visit 2 | - Severe asthma exacerbation during screening or the run-in period |
|  | - ALT <2x ULN, ALP ≤1.5x ULN, bilirubin ≤1.5x ULN at  Visit 1 | - Change in asthma medication (excluding run-in medication and salbutamol provided at Visit 1) |
|  | - Completed all questions in eDiary on ≥4 of the last 7 days of the run-in period | - Laboratory test abnormalities at the discretion of the Investigator |
| **Randomization criteria (randomization)** | - Completed all questions in eDiary on ≥4 of the last 7 days of the stabilization period | - Respiratory infection during the stabilization period expected to affect asthma status or ability participate in the study |
|  |  | - Severe asthma exacerbation during enrollment or stabilization periods |
|  |  | - Change in asthma medication (excluding stabilization period medication provided at Visit 2 and salbutamol provided at Visit 1) |

*ACQ-6 comprises five questions on symptoms and impacts of asthma (ACQ-5) plus rescue use; ^†^if the patient does not meet the reversibility criteria; the assessment may be repeated once within 7 days of Visit 1 if there is a ≥9% increase in FEV_1_ 20−60 minutes following salbutamol or if there is documented evidence of a reversibility assessment within 1 year prior to Visit 1, which demonstrated a post-bronchodilator increase in FEV_1_ of ≥12% and ≥200 mL; ^‡^patients requiring a temporary change in therapy to treat an exacerbation in the 6 weeks prior to Visit 1 may be included at the Investigator's discretion if their condition is stable once they resume their pre-exacerbation maintenance therapy without modification; ^§^patients could be included at the discretion of the Investigator if the benefit of antimuscarinic therapy outweighs the risk and the condition would not contraindicate study participation.

ACQ, Asthma Control Questionnaire; ALT, alanine aminotransferase; ALP, alkaline phosphatase; COPD, chronic obstructive pulmonary disease; ECG, electrocardiogram; eDiary, electronic diary; FEV_1_, forced expiratory volume in 1 second; FVC, forced vital capacity; GOLD, Global Initiative for Chronic Obstructive Lung Disease; ICS, inhaled corticosteroid; LABA, long-acting β_2_-agonist; SABA, short-acting β_2_-agonist; ULN, upper limit of normal

## **Supplementary Table 4.** Study visit schedule for PRO collection in 205832 and 205715

| **Protocol activity** | **Pre-screen** | **Screen**  **run-in** | **Enrollment**  **(beginning**  **of**  **stabilization** | **Treatment period** | | | | | | **Follow-up** | |
| --- | --- | --- | --- | --- | --- | --- | --- | --- | --- | --- | --- |
|  |  |  |  | **Fixed treatment period** | | | | **Variable treatment**  **period** | |  |  |
| **Week** | | | | | | | | | | | |
| 205832 | -6 to -2 | -2 |  | 0  (Baseline) | 4 | 12 | 24 |  |  | EW | Safety  follow-up |
| 205715 | -6 to -7 | -5 | -2 | 0  (Baseline) | 4 | 12 | 24 | 36 | 52 | EW | Safety  follow-up |
| **Visit** | | | | | | | | | | | |
| 205832 | 0 | 1 |  | 2  (Randomization) | 3 | 4 | 5  (Final visit) |  |  | EW | Safety  follow-up |
| 205715 | 0 | 1 | 2 | 3 (Randomization) | 4 | 5 | 6 | 7 | 8 | EW | Safety  follow-up |
| **E-RS: COPD + asthma symptoms (eDiary at home, morning and evening)** | | | | | | | | | | | |
| 205832 |  | X |  | X | X | X | X |  |  | X | X |
| 205715 |  | X | X | X | X | X | X | X | X | X |  |
| **PGI-S** | | | | | | | | | | | |
| 205832 |  |  |  | X | X | X | X |  |  | X |  |
| 205715 |  |  |  | X | X | X | X | X | X | X |  |
| **PGIC** | | | | | | | | | | | |
| 205832 |  |  |  |  | X | X | X |  |  | X |  |
| 205715 |  |  |  |  | X | X | X | X | X | X |  |
| **SGRQ** | | | | | | | | | | | |
| 205832 |  |  |  | X | X | X | X |  |  | X |  |
| 205715 |  |  |  | X |  | X | X | X | X | X |  |
| **AQLQ** | | | | | | | | | | | |
| 205832 |  |  |  | X | X | X | X |  |  | X |  |
| 205715 |  |  |  | X | X | X | X | X | X | X |  |
| **ACQ-5** | | | | | | | | | | | |
| 205832 |  |  |  |  | X | X | X |  |  | X |  |
| 205715 |  | X | X | X | X | X | X | X | X | X |  |

ACQ, Asthma Control Questionnaire; AQLQ, Asthma Quality of Life Questionnaire; eDiary, electronic diary; E-RS: Asthma, Evaluating Respiratory Symptoms questionnaire in Asthma; E-RS: COPD, Evaluating Respiratory Symptoms questionnaire in chronic obstructive pulmonary disease; PGIC, Patient Global Impression of Change; PGI-S, Patient Global Impression of Severity; PRO, patient-reported outcome; SGRQ, St George’s Respiratory Questionnaire

## **Supplementary Table 5.** Summary of clinical trials 205832 and 205715

| **Study** | **205832 (final blinded data)** | **205715 (Interim blinded data)** |
| --- | --- | --- |
| Design | Phase IIb, randomized, double-blind, three-arm parallel-group, efficacy, safety, and tolerability trial | Phase IIIa, randomized, double-blind, active controlled, six-arm, parallel-group efficacy, safety, and tolerability trial |
| Population | Patients with moderate, not well controlled asthma: ACQ-6 total score >0.75 at randomization despite treatment with maintenance ICS  N=421 | Patients with moderate/severe uncontrolled asthma: ACQ-6 score ≥1.5 despite treatment with maintenance therapy (>250 mcg/day FP or equivalent plus LABA)  N=2270 |
| Treatment duration | 24 weeks | 24–52 weeks* |
| PROs | E-RS: COPD  SGRQ  AQLQ  ACQ  Patient global assessments of symptom severity  PGIC | E-RS: COPD  SGRQ  AQLQ  ACQ  WPAI-SHP  Patient global assessments of symptom severity  PGIC |
| Study treatment groups^†^ | UMEC 62.5 mcg  UMEC 31.25 mcg  Placebo  All study arms received background therapy of 100 mcg FF daily | FF/UMEC/VI 100/62.5/25 mcg  FF/UMEC/VI 200/62.5/25 mcg  FF/UMEC/VI 100/31.25/25 mcg  FF/UMEC/VI 200/31.25/25 mcg  FF/VI 100/25 mcg  FF/VI 200/25 mcg |

^*^Treatment duration 24 weeks (efficacy endpoints) with some patients continuing in the study for a maximum of 52 weeks; ^†^all study treatments administered once daily; final blinded data for 205832; blinded sample for analysis of 205715 based on interim data included.

ACQ, Asthma Control Questionnaire; AQLQ, Asthma Quality of Life Questionnaire; FF, fluticasone furoate; FP, fluticasone propionate; ICS, inhaled corticosteroid; LABA, long-acting β_2_-agonist; PGIC, Patient Global Impression of Change; PRO, patient reported outcomes; QD, once daily; SGRQ, St George’s Respiratory Questionnaire; UMEC, umeclidinium; VI, vilanterol; WPAI-SHP, Work Productivity and Activity Impairment-Specific Health Problems Questionnaire

## **Supplementary Table 6.** Confirmatory factor analysis of E-RS: COPD at Week 0 in 205832 and 205715 studies

| **Item** | **205715 (N=2244)*** | | |  | **205832 (N=420)** | | |
| --- | --- | --- | --- | --- | --- | --- | --- |
|  | **Breathlessness loadings^†^** | **Cough and sputum loadings^†^** | **Chest symptoms loadings^†^** |  | **Breathlessness loadings^†^** | **Cough and sputum loadings^†^** | **Chest symptoms loadings^†^** |
| Chest congested (1) | - | - | **0.852** |  | - | - | **0.803** |
| Cough (2) | - | **0.886** | - |  | - | **0.829** | - |
| Mucus/phlegm (3) | - | **0.819** | - |  | - | **0.806** | - |
| Difficulty bring up mucus (4) | - | **0.801** | - |  | - | **0.846** | - |
| Chest discomfort (5) | - | - | **0.966** |  | - | - | **0.958** |
| Tight chest (6) | - | - | **0.954** |  | - | - | **0.948** |
| Breathlessness (7) | **0.901** | - | - |  | **0.897** | - | - |
| Describe breathlessness (8) | **0.829** | - | - |  | **0.813** | - | - |
| Short of breath personal activities (9) | **0.841** | - | - |  | **0.822** | - | - |
| Short of breath indoor activities (10) | **0.940** | - | - |  | **0.938** | - | - |
| Short of breath outdoor activities (11) | **0.914** | - | - |  | **0.931** | - | - |
|  | RS-Total |  |  |  | RS-Total |  |  |
| RS-Breathlessness | **0.901** |  |  |  | **0.847** |  |  |
| RS-Cough and Sputum | **0.796** |  |  |  | **0.786** |  |  |
| RS-Chest | **0.936** |  |  |  | **0.930** |  |  |
|  |  |  |  |  |  |  |  |
| CFI^‡^ | 0.921 |  |  |  | 0.872 |  |  |
| SRMR^§^ | 0.046 |  |  |  | 0.061 |  |  |
| RMSEA^║^ | 0.154 |  |  |  | 0.194 |  |  |

*Interim blinded data only; ^†^factor loadings ≥0.40 appear in bold and are considered acceptable; ^‡^CFI of ≥0.9 indicates good model fit;
^§^SRMR of <0.1 indicates good model fit; ^║^RMSEA of <0.08 indicates good model fit.

CFI, comparative fit index; RMSEA, root mean square error of approximation; SRMR, standardized root mean square residual

## **Supplementary Table 7**. Distribution-based approaches for the E-RS: Asthma

| **E-RS: Asthma items** | **205715*** | | |  | **205832** | | |
| --- | --- | --- | --- | --- | --- | --- | --- |
|  | **Mean (SD) at baseline** | **1/2 SD [4]** | **SEM** |  | **Mean (SD) at baseline** | **1/2 SD [4]** | **SEM** |
| RS-Total | 8.3 (6.305) | 3.152 | 3.372 |  | 7.2 (5.656) | 2.828 | 3.632 |
| RS-Breathlessness | 4.0 (3.279) | 1.640 | 1.665 |  | 3.5 (2.993) | 1.497 | 1.889 |
| RS-Cough & Sputum | 2.2 (1.748) | 0.874 | 1.048 |  | 1.9 (1.553) | 0.776 | 1.113 |
| RS-Chest Symptoms | 2.1 (1.872) | 0.936 | 1.077 |  | 1.8 (1.708) | 0.854 | 1.124 |

^*^Interim blinded data only.

E-RS: Asthma, Evaluating Respiratory Symptoms questionnaire in Asthma; SD, standard deviation; SEM, standard error of measurement

## References

1. National Asthma Education and Prevention Programme NH, Lung and Blood Institute, National Institutes of Health (NIH). Expert Panel Report 3: Guidelines for the diagnosis and management of asthma. <http://www.nhlbi.nih.gov/health-pro/guidelines/current/asthma-guidelines>. Accessed 28 April 2020.
2. Global Initiative for Chronic Obstructive Lung Disease (2021) Global strategy for the diagnosis, management and prevention of chronic obstructive pulmonary disease. <https://goldcopd.org/wp-content/uploads/2020/11/GOLD-REPORT-2021-v1.1-25Nov20_WMV.pdf>. Accessed 4 February 2021.
3. Quanjer PH, Stanojevic S, Cole TJ, et al. (2012) Multi-ethnic reference values for spirometry for the 3-95-yr age range: the global lung function 2012 equations. Eur Respir J 40:1324-1343.
4. Norman GR, Sloan JA, Wyrwich KW (2003) Interpretation of changes in health-related quality of life: the remarkable universality of half a standard deviation. Med Care 41:582-592.
